# Association between gut microbiota and diabetic microvascular complications: a two-sample Mendelian randomization study

**DOI:** 10.3389/fendo.2024.1364280

**Published:** 2024-08-02

**Authors:** Peipei Zhou, Zhenning Hao, Yu Chen, Ziqi Zhang, Weilong Xu, Jiangyi Yu

**Affiliations:** ^1^ Department of Endocrinology, Jiangsu Province Hospital of Chinese Medicine, Affiliated Hospital of Nanjing University of Chinese Medicine, Nanjing, China; ^2^ The First Clinical Medical College, Nanjing University of Chinese Medicine, Nanjing, China

**Keywords:** diabetic kidney disease, diabetic microvascular complications, diabetic neuropathy, diabetic retinopathy, Mendelian randomization

## Abstract

**Background:**

Gut microbiota (GM) homeostasis in the human body is closely associated with health, which can be used as a regulator for preventing the onset and progression of disease. Diabetic microvascular complications bring about not only a huge economic burden to society, but also miserable mental and physical pain. Thus, alteration of the GM may be a method to delay diabetic microvascular complications.

**Objective:**

A two-sample Mendelian randomization (MR) analysis was conducted to reveal the causal inference between GM and three core diabetic microvascular complications, namely, diabetic kidney disease (DKD), diabetic retinopathy (DR), and diabetic neuropathy (DNP).

**Methods:**

First, genome-wide association study (GWAS) summary statistics for GM from the MiBioGen consortium and three main diabetic microvascular complications acquired from the FinnGen research project were assessed. Second, a forward MR analysis was conducted to assess the causality of GM on the risk of DKD, DR, and DNP. Third, a series of sensitivity studies, such as heterogeneity tests, pleiotropy evaluations, and leave-one-out analyses, were further conducted to assess the accuracy of MR analysis. Finally, Steiger tests and reverse MR analyses were performed to appraise the possibility of reverse causation.

**Results:**

A total of 2,092 single-nucleotide polymorphisms related to 196 bacterial traits were selected as instrumental variables. This two-sample MR analysis provided strongly reasonable evidence that 28 genetically predicted abundance of specific GM that played non-negligible roles in the occurrence of DKD, DR, and DNP complications were causally associated with 23 GM, the odds ratio of which generally ranged from 0.9 to 1.1. Further sensitivity analysis indicated low heterogeneity, low pleiotropy, and high reliability of the causal estimates.

**Conclusion:**

The study raised the possibility that GM may be a potential target to prevent and delay the progression of diabetic microvascular complications. Further experiments of GM therapy on diabetic microvascular complications are warranted to clarify their effects and specific mechanisms.

## Introduction

Diabetic microvascular complications are characterized by the damage of small vessels or nerves as a result of chronic persistent hyperglycemic state in patients with diabetes mellitus (DM), manifested as abnormal structure and changes in functions of the corresponding targeted organs ultimately (1–3). It is known that diabetic kidney disease (DKD), diabetic retinopathy (DR), and diabetic neuropathy (DNP) are three major diabetic chronic microvascular complications that need to be screened comprehensively upon diagnosis of type 2 diabetes (T2D) and type 1 diabetes (T1D) in the fifth year and even at least annually thereafter because of the characteristics of insidious onset and irreversible progression, which leads to enormous economic burden and prolonged potential physical and mental suffering (4, 5). In spite of the large number of novel treatments available, the incidence and prevalence of DM continue to increase around the world and show a trend of younger generations being affected, which leads to an obvious rise in the corresponding microvascular complications (6–8).

Plenty of microbes are enriched in the gastrointestinal tract, the biggest microbiota habitat in the human body; meanwhile, these microbiota exist in a dynamic balanced state for health regulation purposes (9). The composition and metabolism of gut microbiota (GM) play an important role in DM and its complications (10, 11), which is affected by multiple factors such as diet (12), demographics (13), and use of medication (14). Recent studies have focused on the association between GM and DM and its microvascular complications and especially put forward the theory of “gut–kidney axis” (15), “gut–retina axis” (16), “gut–brain axis” (17), and “gut–peripheral nerve axis” (18). Therefore, focusing on the modulation of GM with probiotics, prebiotics, synbiotics, or even fecal microbial transplantation may be a promising breakthrough direction on DM and subsequent microvascular complications. Nonetheless, the link between GM and diabetic microvascular complications driven by causative mechanistic interactions or merely being correlative remains unclear.

Mendelian randomization (MR) is a complementary statistical approach that leverages the genetic variants associated with exposure factors such as instrumental variables (IVs) to imply the causal inference between exposure and disease outcomes (19). MR analyses for inferring the causal relationship of GM on multiple diseases have been widely applied due to various findings from large-scale genome-wide association studies (GWASs) to data conducted on GM (20, 21). Previous studies have indicated that some GM are causally associated with T1D (22) and T2D (23). However, there was no evidence that demonstrated whether GM became a potential causal factor on diabetic microvascular complications. Therefore, MR analysis allows us to assess the contribution of GM on diabetic microvascular complications in the present study. Furthermore, this study could facilitate drug discovery and obtain reliable surrogate biomarkers to predict the onset and progression of diabetic microvascular complications, including DKD, DR, and DNP.

## Materials and methods

### Data sources

Genetic variants for GM were obtained from the MiBioGen consortium, which performed the largest multi-ancestry genome-wide meta-analysis published to date (20). The study included 18,340 individuals, of whom over 70% were of European ancestry in 24 cohorts, targeting three distinct variable regions of the 16S rRNA gene to profile the microbial composition and utilizing direct taxonomic binning for conducting taxonomic classification. In addition, every sample was rarefied to 10,000 reads in all datasets on the interpretation of different sequencing depths. Microbiome quantitative trait loci mapping analysis, including 211 taxa (five levels, in the order of genus, family, order, class, and phylum), was conducted to identify the effect of host genetics on the relative abundance levels of microbial taxa. More details related to the GM data could be found elsewhere (20). All the GWAS summary statistics of diabetic complications in this study were acquired from the FinnGen research project (https://r9.finngen.fi/). Lastly, a total of 4,111 cases and 308,539 controls in DKD, 10,413 cases and 308,633 controls in DR, and 2,843 cases and 271,817 controls in DNP were included.

### Ethics statement

The summary-level data involved in this study are free and publicly available for download. The respective institutions have approved the ethics statement of each GWAS in this study. There were no individual-level data in this study; thus, new ethical review board approval was unnecessary.

### Instrumental variable selection

The selection of optimal IVs was vital for the robustness and accuracy of the causal association, which conformed to the MR’s three principal assumptions (24), specifically for relevance, independence, and exclusion assumption. To explore more relations, a relatively more comprehensive threshold (*p* < 1e−05) of single-nucleotide polymorphisms (SNPs) associated with GM was applied (25). Data of the European-based 1,000 Genome Projects were set as the reference panel for performing a linkage disequilibrium (LD) analysis, where SNPs had *r* ^2^ < 0.001 and the window size was 10,000 kb. *F* statistics not less than 10 represented the notable strength of the selected SNPs for each bacterial taxon, the equation of which is *R* ^2^(*N* − 2)/(1 − *R* ^2^) (26), where *R* ^2^ denotes the proportion in exposure variance of each selected IV interpretation and *N* represents the sample size (27). Minor allele frequency (MAF) <0.01 of SNPs was removed, aiming at clearing mutations in less than 1% of the population. Furthermore, palindromic SNPs were removed to prevent distortion of strand orientation or allele coding.

### Statistical analysis

Inverse variance weighted (IVW) was the primary method to examine the causal association between GM and diabetic complications based on ratio estimates of each variant (28), which provided a more conservative but robust estimate (29), the *p*-value of which determined the criterion for the existence of a causal association between exposure and outcome. Cochran’s *Q* test was used for the assessment of the heterogeneity among IVs, and the random-effects model was applied in the presence of significant heterogeneity; otherwise, the fixed-effects model was used (30). A series of additional MR analyses were conducted for calculating the causal effect values, including the weighted median, MR-Egger regression, simple mode, and weighted mode methods. The weighted median method, the median of the weighted ratio estimates of valid variants as the total weight of the instrument, showed consistent results with IVW in the condition of even up to 50% of invalid IVs (28). The MR-Egger regression test employed a weighted linear regression instead of setting the intercept to zero in IVW and allowed the presence of over 50% of invalid IVs, intercept estimated by MR-Egger regression could serve to estimate the average horizontal effect of pleiotropy (31, 32). The largest cluster of SNPs was applied in simple mode and the weights were assigned to each SNP in weighted mode (33, 34). In scenarios where the beta values for exposure and outcome summary data exhibited significant disparity in distribution, the correct factor will be utilized.

Evaluation of overall horizontal pleiotropy was conducted by the MR pleiotropy residual sum and outlier (MR-PRESSO) global test, and then outlier removal could correct this pleiotropy (25). The intercept from the MR-Egger test further verified the sensitivity; *p*_intercept_ < 0.05 indicated horizontal pleiotropy (31). Directional causal inference judged by the MR Steiger directionality test was made (35). Additionally, the leave-one-out analysis was performed to validate data robustness and avoid affecting significant results via a single SNP (36).

### Reverse Mendelian randomized analysis

A reverse MR analysis was also conducted to explore whether the disease outcomes have any causal impact on the GM, especially the identified significant ones. Noteworthily, SNPs related to each genus of diabetic complications at the locus-wide significance threshold (*p* < 5e−08) were selected as potential IVs to obtain more comprehensive results (22), which were different from screening the IVs in the pre-MR analysis.

The threshold of statistical significance was identified as *p* < 0.05 and odds ratio (OR) with 95% confidence interval (CI) was regarded as the effect between GM and diabetic complications. False discovery rate (FDR) correction was conducted; a *q*-value of more than 0.1 means no suggestive causal association (37). R software (version 4.3.1) was used for all the above statistical analyses. We used the Strengthening the Reporting of Observational Studies in Epidemiology using Mendelian Randomization (STROBE-MR) checklist published in 2021 as reference (38).

R software (version 4.3.1. R Foundation for Statistical Computing 2023) was utilized in all of the analyses. The packages TwoSampleMR (version 0.5.7), MRPRESSO (version 1.0), vroom (version 1.6.4), grid (version 4.3.1), forestploter (version 1.1.1), data.table (version 1.14.8), phenoscanner (version 1.0), dplyr (version 1.1.2), fdrtool (version 1.2.17), plinkbinr (version 0.0.0.9000), and ieugwasr (version 0.1.5) were used.

## Results

### Causal effects of GM on diabetic microvascular diseases

**Figure 1:**
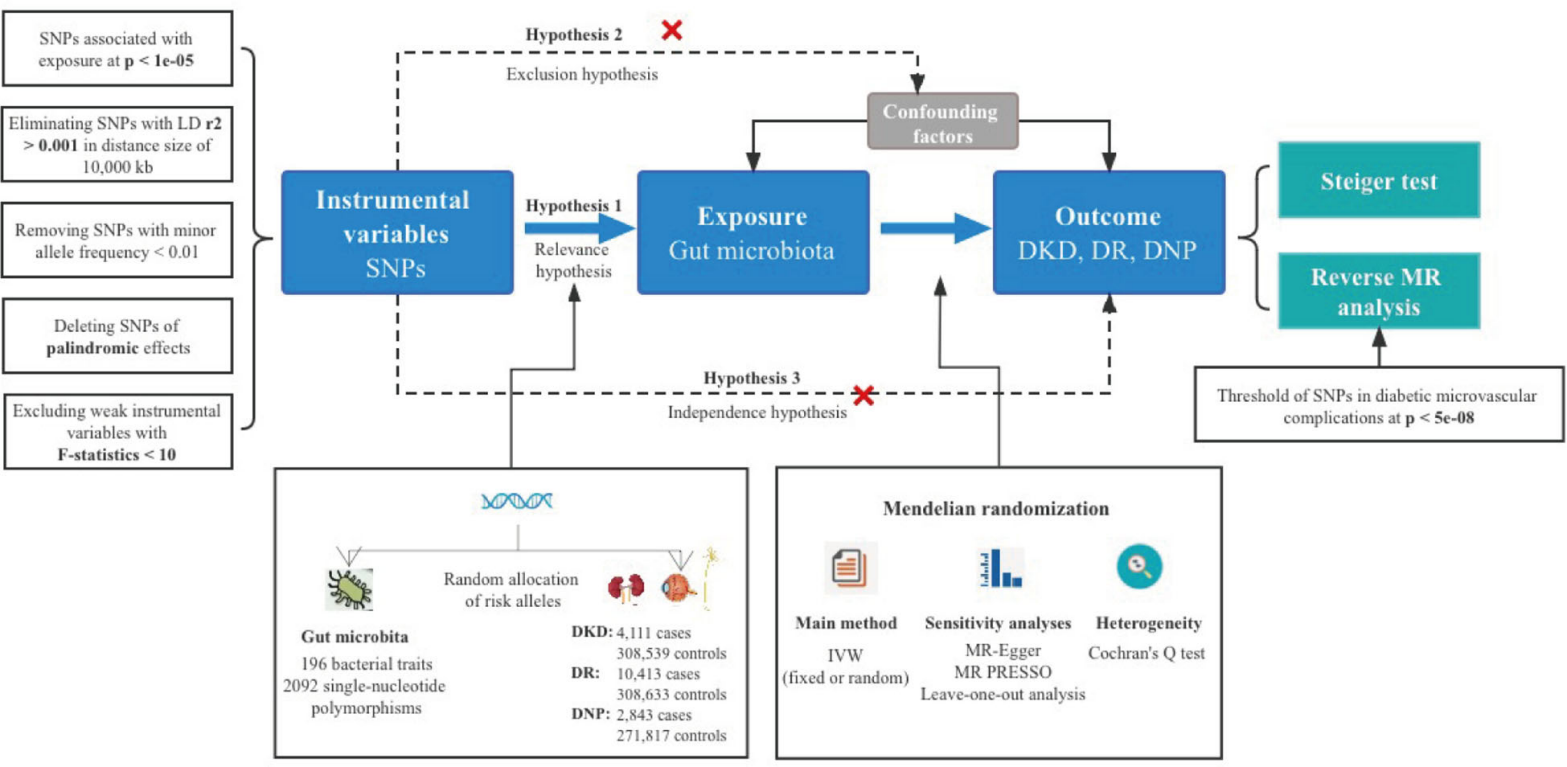
Overview of MR analysis process and major hypotheses.

A total of 196 bacterial taxa were identified for MR analysis after removing 15 unknown families or genera. All the *F* statistics of the IVs selected were more than 10, indicating no evidence of weak instrumental bias. Meanwhile, over three SNPs of each GM were included for a successful MR-PRESSO test. In addition, the MAFs were all more than 0.01. Lastly, a total of 2,092 IVs were identified, including 9 phyla (114 SNPs), 16 classes (179 SNPs), 20 orders (216 SNPs), 32 families (352 SNPs), and 119 genera (1,231 SNPs). Detailed information of IVs used in the MR analysis for the causal inference is presented in **Supplementary Table S1**. It was worth noting that the more taxonomically distinct GM was chosen when GM shared the same SNPs; in other words, we would pick the class *Verrucomicrobiae* instead of the order *Verrucomicrobiales*. An overall view of the MR analysis process and major hypotheses is shown in **Figure 1**.

### Diabetic kidney disease

In the phylum level, only the genetic predicted Bacteroidetes (OR = 1.43, 95% CI = 1.09–1.88, *p* = 1.08e−02) was causally associated with DKD. As for the class level, we found that the higher genetically predicted Bacteroidia (OR = 1.45, 95% CI = 1.12–1.87, *p* = 4.57e−03) and Verrucomicrobiae (OR = 1.40, 95% CI = 1.13–1.73, *p* = 1.80e−03) were identified as higher risks of DKD. Meanwhile, the genetically predicted genera *Catenibacterium* (OR = 1.31, 95% CI = 1.08–1.59, *p* = 6.40e−03), *Lachnoclostridium* (OR = 1.43, 95% CI = 1.13–1.82, *p* = 3.11e−03), and *Parasutterella* (OR = 1.27, 95% CI = 1.07–1.51, *p* = 6.52e−03) were also causally associated with DKD. However, we found that family *Bacteroidaceae* (OR = 0.72, 95% CI = 0.52–0.99, *p* = 4.64e−02), family *Victivallaceae* (OR = 0.87, 95% CI = 0.77–0.98, *p* = 2.45e−02), genus *Coprococcus2* (OR = 0.74, 95% CI = 0.58–0.96, *p* = 2.47e−02), and genus *Lactococcus* (OR = 0.85, 95% CI = 0.73–0.99, *p* = 3.93e−02) played protective roles in the causal inference between GM and DKD.

### Diabetic retinopathy

We found that the genetically predicted class Bacteroidia was causally associated with DR (OR = 1.24, 95% CI = 1.05–1.46, *p* = 9.75e−03). In the family level, the higher genetically predicted *BacteroidalesS24* (OR = 1.16, 95% CI = 1.02–1.33, *p* = 2.45e−02), *ClostridialesvadinBB60group* (OR = 1.18, 95% CI = 1.06–1.32, *p* = 2.62e−03), and *Peptostreptococcaceae* (OR = 1.17, 95% CI = 1.03–1.33, *p* = 1.90e−02) were related to the higher abundance of DR. In addition, IVW results demonstrated a harmful effect of the host-genetic-driven increase in the genera *Eubacterium nodatum group* (OR = 1.08, 95% CI = 1.01–1.17, *p* = 3.53e−02), *Actinomyces* (OR = 1.15, 95% CI = 1.01–1.32, *p* = 3.25e−02), *Olsenella* (OR = 1.10, 95% CI = 1.01–1.20, *p* = 2.16e−02), *Parasutterella* (OR = 1.12, 95% CI = 1.01–1.25, *p* = 3.87e−02), *RuminococcaceaeUCG003* (OR = 1.15, 95% CI = 1.00–1.32, *p* = 4.54e−02), and *RuminococcaceaeUCG011* (OR = 1.15, 95% CI = 1.04–1.28, *p* = 5.83e−03) on the risk of DKD, except for the genus *Eisenbergiella* (OR = 0.90, 95% CI = 0.82–0.99, *p* = 3.17e−02) acting as a protective factor.

### Diabetic neuropathy

The genetic liability for the family *Acidaminococcaceae* (OR = 0.62, 95% CI = 0.46–0.84, *p* = 1.76e−03), family *Peptococcaceae* (OR = 0.70, 95% CI = 0.54–0.90, *p* = 5.65e−03), and genus *Eubacterium coprostanoligenes group* (OR = 0.68, 95% CI = 0.50-0.93, *p* = 1.61e−02) contributed to a decreased abundance of DNP in the results of IVW analyses. Nevertheless, the higher genetically predicted genera *Alistipes* (OR = 1.65, 95% CI = 1.18–2.31, *p* = 3.21e−03), *ChristensenellaceaeR_7group* (OR = 1.52, 95% CI = 1.03–2.23, *p* = 3.28e−02), *Eggerthella* (OR = 1.28, 95% CI = 1.05–1.55, *p* = 1.42e−02), and *RuminococcaceaeUCG013* (OR = 1.35, 95% CI = 1.01–1.82, *p* = 4.57e−02) were causally associated with a higher abundance of DNP.

**Figure 2:**
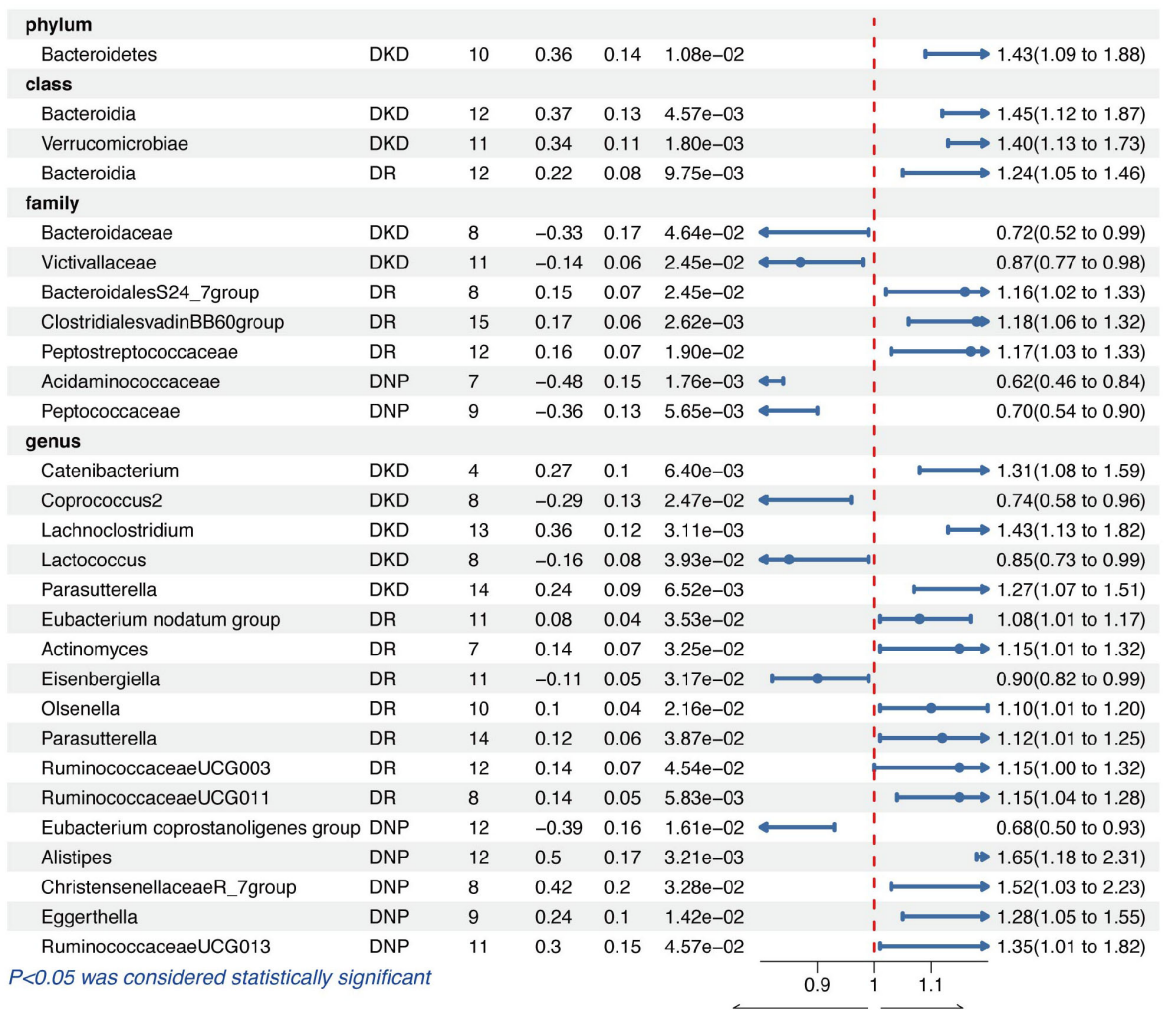
The results of the IVW method for causal inference of GM on diabetic microvascular complications.

The significant results of the IVW analysis for the causal inference of GM on diabetic microvascular complications are presented in **Figure 2**, where fixed-effect models were applied. No significant *q*-value of the GM was discovered in FDR correction analysis. Results of all MR analyses (*p*-value of IVW method < 0.05) along with FDR correction for the IVW method are shown in **Supplementary Tables S2**–**S4** and visual inspection of MR analyses is shown in **Supplementary Figures S1**–**S3**.

### Sensitivity analysis

No evidence of heterogeneity (*p* > 0.05) was observed in Cochran’s *Q* test, thus resulting in the fixed-effect models being used for the causal inference in the IVW analyses. Moreover, no significant horizontal pleiotropy existed in the MR-PRESSO analysis (global test *p* > 0.05), and MR-Egger regression intercept analysis was further verified (*p* > 0.05). Therefore, we could conclude that the results of IVW were authentic in the absence of pleiotropy and heterogeneity. At the same time, the Steiger test indicated no directional causal estimations between GM and diabetic microvascular complications. Summary data of heterogeneity, pleiotropy, and direction analyses are presented in **Table 1**. The leave-one-out analysis indicated that no single SNP affects the causal inference of GM on diabetic microvascular complications (**Supplementary Figures S4**–**S6**).

**Table 1:** Heterogeneity, pleiotropy and directional analyses of GM on diabetic microvascular complications.

Level	Exposure	Outcome	Heterogeneity P for Cochran's Q	MRegger_intercept	Horizontal pleiotrophy P for Egger intercept	steiger_pval	MRPRESSO
phylum	*Bacteroidetes*	DKD	0.848	-0.003	0.907	3.33E-65	0.865
class	*Bacteroidia*	DKD	0.906	-0.002	0.914	3.26E-73	0.92
class	*Verrucomicrobiae*	DKD	0.451	0.034	0.263	1.16E-51	0.481
family	*Bacteroidaceae*	DKD	0.284	-0.021	0.738	3.60E-34	0.338
family	*Victivallaceae*	DKD	0.710	0.000	0.996	5.98E-56	0.718
genus	*Catenibacterium*	DKD	0.902	-0.103	0.586	3.73E-19	0.911
genus	*Coprococcus2*	DKD	0.701	0.042	0.590	7.31E-34	0.745
genus	*Lachnoclostridium*	DKD	0.707	0.005	0.863	1.87E-52	0.733
genus	*Lactococcus*	DKD	0.782	0.046	0.389	5.40E-39	0.823
genus	*Parasutterella*	DKD	0.952	0.018	0.385	1.02E-68	0.941
class	*Bacteroidia*	DR	0.865	-0.013	0.338	1.68E-73	0.865
family	*BacteroidalesS24_7group*	DR	0.359	0.009	0.758	1.61E-39	0.415
family	*ClostridialesvadinBB60group*	DR	0.720	0.017	0.238	1.51E-72	0.749
family	*Peptostreptococcaceae*	DR	0.780	0.027	0.051	1.10E-72	0.761
genus	*Eubacterium nodatum group*	DR	0.889	-0.023	0.381	3.48E-53	0.896
genus	*Actinomyces*	DR	0.791	-0.015	0.442	1.09E-33	0.821
genus	*Eisenbergiella*	DR	0.425	-0.008	0.852	2.09E-49	0.466
genus	*Olsenella*	DR	0.330	0.028	0.156	3.34E-47	0.385
genus	*Parasutterella*	DR	0.963	0.004	0.787	1.61E-69	0.96
genus	*RuminococcaceaeUCG003*	DR	0.629	0.013	0.476	1.55E-58	0.631
genus	*RuminococcaceaeUCG011*	DR	0.208	0.029	0.441	2.54E-39	0.249
family	*Acidaminococcaceae*	DNP	0.551	-0.078	0.148	1.11E-30	0.595
family	*Peptococcaceae*	DNP	0.804	-0.003	0.940	6.43E-48	0.816
genus	*Eubacterium coprostanoligenes group*	DNP	0.676	-0.008	0.828	1.72E-50	0.68
genus	*Alistipes*	DNP	0.663	0.039	0.444	5.65E-46	0.691
genus	*ChristensenellaceaeR_7group*	DNP	0.426	-0.019	0.746	5.20E-32	0.477
genus	*Eggerthella*	DNP	0.504	-0.032	0.536	1.50E-40	0.502
genus	*RuminococcaceaeUCG013*	DNP	0.847	-0.022	0.534	2.80E-53	0.854

### Reverse causal effects of diabetic microvascular diseases on GM

When diabetic microvascular complications were set as exposure and GM as outcome, a total of 6 IVs on DKD, 17 IVs on DR, and 4 IVs on DNP were included according to the strict quality selection (**Supplementary Table S5**).

**Figure 3:**
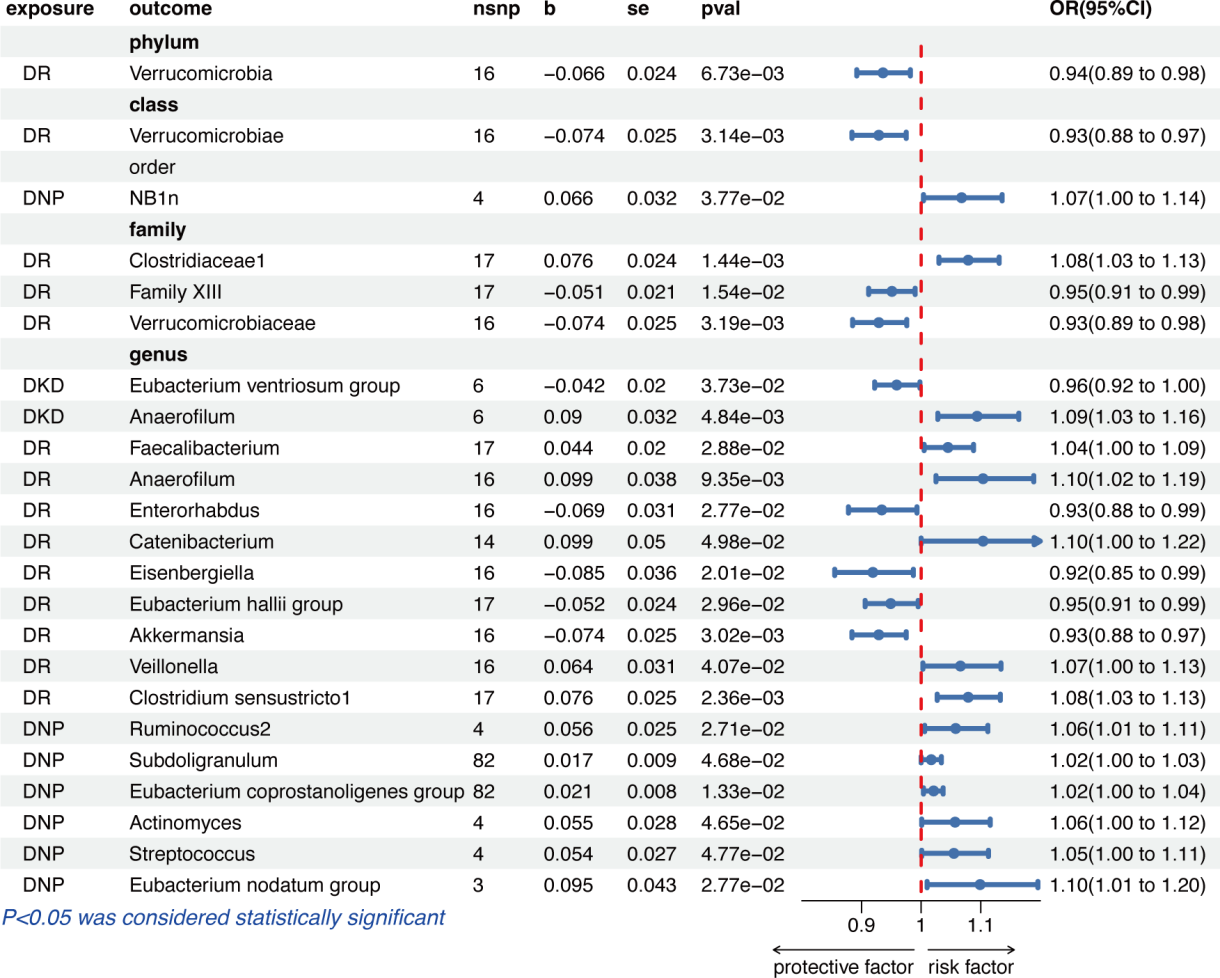
The results of the IVW method given as odds ratio (OR) and 95% confidence interval (CI) for causal inference of diabetic microvascular complications on GM. DR, diabetic retinopathy; DNP, diabetic neuropathy; DKD, diabetic kidney disease.

**Figure 3** shows that genetically predicted diabetic microvascular complications were causally associated with some other GM in the IVW results. Summary data of MR analysis between DKD, DR, DNP, and GM are separately presented in **Supplementary Tables S6** and **S7**. DKD was a protective factor for *Eubacterium ventriosum group* (OR = 0.96, 95% CI = 0.92–1.00, *p* = 3.73e−02) and a risk factor for *Anaerofilum* (OR = 1.09, 95% CI = 1.03–1.16, *p* = 4.84e−03) in the genus level.

The IVW results indicated that a higher genetically predicted DR, on the one hand, had a beneficial role on the phylum *Verrucomicrobia* (OR = 0.94, 95% CI = 0.89–0.98, *p* = 6.73e−03), the class *Verrucomicrobiae* (OR = 0.93, 95% CI = 0.88–0.97, *p* = 3.14e−03), the families *Family XIII* (OR = 0.95, 95% CI = 0.91–0.99, *p* = 1.54e−02) and *Verrucomicrobiaceae* (OR = 0.93, 95% CI = 0.89-0.98, *p* = 3.19e−03), and the genera *Enterorhabdus* (OR = 0.93, 95% CI = 0.88–0.99, *p* = 2.77e−02), *Eisenbergiella* (OR = 0.92, 95% CI = 0.85–0.99, *p* = 2.01e−02), *Eubacterium hallii group* (OR = 0.95, 95% CI = 0.91–0.99, *p* = 2.96e−02), and *Akkermansia* (OR = 0.93, 95% CI = 0.88–0.97, *p* = 3.02e−03); on the other hand, DR had an adverse effect on the family *Clostridiaceae1* (OR =1.08, 95% CI = 1.03-1.13, *p* = 1.44e−03) and the genera *Faecalibacterium* (OR =1.04, 95% CI = 1.00–1.09, *p* = 2.88e−02), *Anaerofilum* (OR =1.10, 95% CI = 1.02–1.19, *p* = 9.35e−03), *Catenibacterium* (OR =1.10, 95% CI = 1.00–1.22, *p* = 4.98e−02), *Veillonella* (OR =1.07, 95% CI = 1.00–1.13, *p* = 4.07e−02), and *Clostridium sensustricto1* (OR =1.08, 95% CI = 1.03–1.13, *p* = 2.36e−03).

In the order level, DNP was causally associated with NB1n (OR =1.07, 95% CI = 1.00–1.14, *p* = 3.77e−02). As for the biological genus classifications, the IVW results indicated that DNP was causally associated with *Ruminococcus2* (OR =1.06, 95% CI = 1.01–1.11, *p* = 2.71e−02), *Subdoligranulum* (OR =1.02, 95% CI = 1.00–1.03, *p* = 4.68e−02), *Eubacterium coprostanoligenes group* (OR =1.02, 95% CI = 1.00–1.04, *p* = 1.33e−02), *Actinomyces* (OR =1.06, 95% CI = 1.00–1.12, *p* = 4.65e−02), *Streptococcus* (OR =1.05, 95% CI = 1.00–1.11, *p* = 4.77e−02), and *Eubacterium nodatum group* (OR = 1.10, 95% CI = 1.01–1.20, *p* = 2.77e−02).

Cochrane’s *Q* test, MR-PRESSO, and MR-Egger regression intercept analysis further demonstrated that no evidence of heterogeneity and pleiotropy existed. Meanwhile, the Steiger test showed no directional causal estimations. Summary data of heterogeneity, pleiotropy, and direction analyses are presented in **Supplementary Table S8**.

## Discussion

To the best of our knowledge, this is the first MR study to evaluate the causal inferences between diabetic microvascular complications and GM from a genetic perspective using the summary statistics of diseases from the FinnGen consortium R9 release data and GM from the largest GWAS meta-analysis conducted by the MiBioGen consortium. Our research mainly discussed the diabetic microvascular complications and the published article discussed six complications (including acute complications of diabetes). Although we chose the same GWAS summary data, we have more cases and controls with DR compared with those in the article of PMID 38481313 (39). Moreover, we conducted the Steiger test and reverse MR analysis to explore the genetically predicted GM on the diabetic microvascular complications.

**Figure 4:**
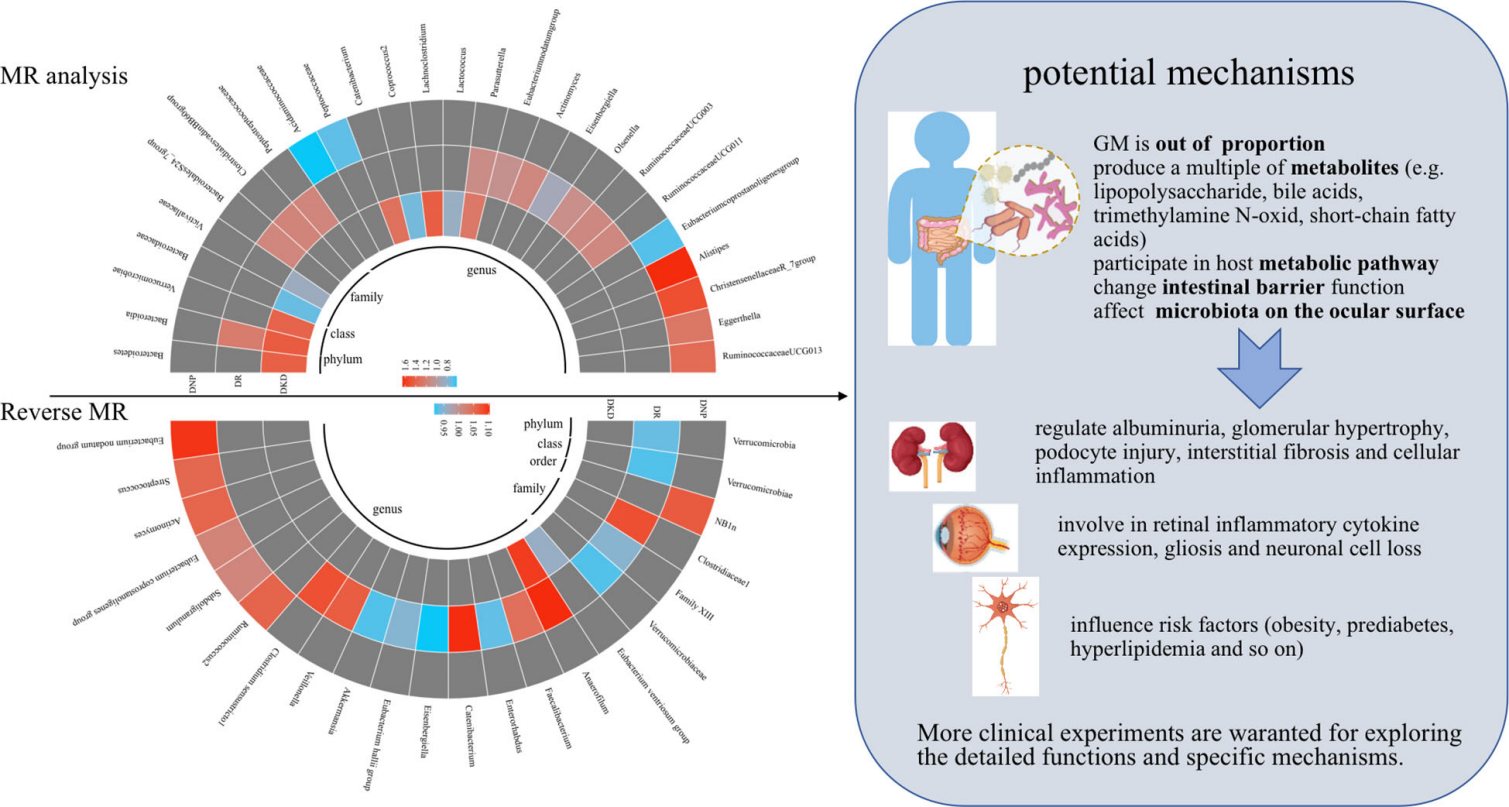
Visual results of the causality inference between GM and diabetic microvascular complications were shown on the left and potential mechanisms were presented on the right. Red represents risk factors, blue represents protective factors, and gray represents negative causal inference between disease and GM. MR, Mendelian randomization.

This two-sample MR analysis provided reasonable evidence that 28 genetically predicted abundance of specific GM play non-negligible roles in the occurrence of DKD, DR, and DNP. Similarly, reverse MR analysis indicated that genetic liability to three chronic microvascular complications was causally associated with 23 GM. In particular, the IVW method indicated a bi-directional causal relationship between *Eisenbergiella* and DR. Amazingly, *Eubacterium coprostanoligenes group* and DNP presented contradictory effects on the causal inference via IVW analysis. These results could bring about implications for new effective treatments to counter DM-associated chronic microvascular complications. Visual results of the causality inference between GM and diabetic microvascular complications are shown in **Figure 4**.

DKD, a devastating complication of T1D and T2D and a leading cause of end-stage renal disease, occurs in 20% to 50% of patients with DM (40). Few treatment options were available to better stop or delay the onset and progression of DKD. In our study, the family *Bacteroidaceae* showed an opposite causal inference from its phylum and class levels. Although *Catenibacterium*, *Lachnoclostridium*, *Coprococcus2*, and *Lactococcus* belonged to Firmicutes, they presented different causal relationships on DKD. Previous studies (41, 42) indicated that the relative abundance of Bacteroidetes decreased and that of Firmicutes increased in DKD compared with healthy individuals and in DM without kidney diseases, which was partly consistent with our results. Abnormal ratios of Bacteroidetes/Firmicutes were decreased in rats with chronic kidney disease compared to controls, which was related to increased acetate- and butyrate-producing bacteria (43). This discrepancy could be attributed to different species, complicated diet habits, and the living environment of people. Our results also found that Verrucomicrobiae and *Parasutterella* possibly played harmful roles on DKD, whereas *Victivallaceae* had suggestive protective effects on DKD. A translational human study (44) indicated that *Parasutterella* abundance was positively associated with obesity and T2D, where fatty acid biosynthesis pathway and L-cysteine might be relevant. Similar to the analysis of GM on patients with DKD, the abundance of Verrucomicrobia and Proteobacteria was relatively increased in patients with DKD in comparison with healthy individuals (45).

Furthermore, a multitude of metabolites produced by GM are essential mediators in the crosstalk between the microbial and host environment, such as lipopolysaccharide, bile acids, trimethylamine N-oxide, and short-chain fatty acids, regulating albuminuria, glomerular hypertrophy, podocyte injury, interstitial fibrosis, and cellular inflammation (46–48).

Bacteroides participated in the synthesis of fatty acids, such as acetate, propionate, and butyrate (49, 50). Olfr-78 receptors did not respond to butyrate but were more sensitive to propionate and acetate. Acetate was in the dysregulation of the dynamic homeostasis of fatty acid metabolism by activating Olfr-78 receptors, which led to tubulointerstitial injury in DKD (51). This might be a potential mechanism by which bacillus-like organisms affect diabetic nephropathy.

DR is the dominant cause of preventable blindness in adults and identified in a third of people with DM (52). Five years after the diagnosis of DM, the number of children and adolescents who progressed to DR increased rapidly (53); thus, annual screening with fundus photography and regulation of GM from diet or lifestyle are especially necessary. Substantial microorganisms are enriched not only on the intestinal but also on the ocular surface. The ocular flora plays an important role in the regulation of ocular immunity and prevention of pathogens, especially in the conjunctiva and cornea (54). Studies have shown that patients with diabetes with diabetic complications have higher conjunctival flora than patients with type 2 diabetes without complications (55). Moreover, studies discovered that the primary composition of microbiota on the ocular surface is Proteobacteria and Actinobacteria (56), accounting for over 87% of all microorganisms that exist in the eye along with Firmicutes (57). Modulation of microbiota through oral feeding of *Lactobacillus paracasei* secreting Ang-(1-7) could reduce retinal inflammatory cytokine expression and retinal gliosis and block neuronal cell loss (58).

Our results concluded that Bacteroidetes (Bacteroidia and *BacteroidalesS24*), Firmicutes (including *ClostridialesvadinBB60group*, *Peptostreptococcaceae*, and *Eubacterium nodatum group*), Actinobacteria (including *Actinomyces* and *Olsenella*), *Parasutterella*, and Verrucomicrobia (including *RuminococcaceaeUCG011* and *RuminococcaceaeUCG003*) were the potentially detrimental bacteria, except for *Eisenbergiella*, acting as the latent protective bacteria on the occurrence and progression of DR via causal inference. Beli et al. discovered a significant increase of Bacteroidetes and Verrucomicrobia on DR with the decrease of acellular capillaries and leukocyte infiltration (59), which is similar to our results. Moreover, Firmicutes was a risk microbiota in the development of DR. The difference from our results was possibly attributed to the mouse models used, being kept in a sterile environment, and fixed diets. Different from a previously reported MR analysis (60), the results of our study were more comprehensive because we chose the updated data in the FinnGen research project and we all agreed that *RuminococcaceaeUCG011* was a risk factor for the occurrence and progression of DR. Additionally, IVs are enough for us to conduct reverse MR analyses in our study. Interestingly, we discovered that *Parasutterella* played a harmful role on the progression of DKD and DR considering the possible reasons of change in fatty acid biosynthesis and L-cysteine in patients with DM (44). However, whether there is a specific reason for their progression other than DNP remains to be further explored.

DNP, which damages the diffuse and focal nervous system, is the most common complication occurring in up to 50% of individuals with DM, and distal symmetric polyneuropathy is the main characteristic (61). It can also affect other organs, resulting in cardiac autonomic neuropathy accompanied by weakness and orthostatic tachycardia, gastrointestinal autonomic dysfunction including early satiety with poor appetite and nausea, esophageal dysfunction with difficulty swallowing, bladder dysfunction, and sudomotor autonomic disturbance, among others (62). The current treatment of DNP is limited, and corresponding studies on GM that explore the ideal intervention targets and preventive strategies are on the rise. Our results indicated that the lower abundance of *Acidaminococcaceae*, *Peptococcaceae*, and *Eubacterium coprostanoligenes group*, and the higher abundance of *Alistipes*, *ChristensenellaceaeR_7group*, *Eggerthella*, and *RuminococcaceaeUCG013* were causally associated with appearance and deterioration of DNP. Ma et al. discovered no significance of high blood glucose and painful hypersensitivity in animals induced by streptozotocin as major depletion of GM in comparison with controls (63). However, as far as we know, there are no relevant studies observed between our chosen GM and DNP, but they are associated with relative risk factors. The relationship between gut dysbiosis, neuronal damage, and dyskinesia is not yet fully understood, but GM clearly plays an important role in maintaining the function of the enteric nervous system (64). In a recent study, Nyavor et al. (65) found a reduction in inhibitory neuromuscular transmission and a loss of inhibitory motor neurons in muscles in rats fed a high-fat diet. High-fat diets also lead to microbiological imbalances in the bacterial flora, such as increased numbers of *Aspergillus*, *Lactobacillus*, and *Bifidobacterium*. These changes are associated with neuropathy and intestinal motility disorders.

Insulin resistance is closely linked to the onset and progression of DNP (66, 67). Yuan et al. indicated that Firmicutes was decreased and Bacteroidetes was increased in insulin-resistant subjects compared with insulin-sensitive individuals (68). *Acidaminococcaceae* and *Peptococcaceae* belonged to Firmicutes, whereas *Alistipes* was part of Bacteroidetes. Furthermore, *Peptococcaceae* was significantly more prevalent and *Alistipes* was less prevalent in individuals with insulin resistance via influencing serum concentrations of angiopoietin-like 4 and adropin, which was consistent with our causal inference of GM on DNP. *ChristensenellaceaeR_7group* was positively correlated with obesity and would be a potential therapeutic target of traditional Chinese medicine to relieve obesity (69). Although no research observed the relationship between *RuminococcaceaeUCG013* and DNP, a study demonstrated that abnormalities of *Ruminococcaceae* caused cognitive dysfunction in DNP rats (70). In general, the causality of GM found in our study needs to be further verified.

The above positive strains showed no causal inference in reverse MR analyses except for *Eisenbergiella* and *Eubacterium coprostanoligenes group*. *Eisenbergiella* showed a bi-directional causal relationship in the reverse MR analysis. A plethora of studies revealed that alterations of the abundance of *Eisenbergiella* led to numerous diseases, including multiple sclerosis, rheumatoid arthritis, and autism (71–73). Upregulated *Eisenbergiella* affected fatty acid metabolism by the production of short-chain fatty acids to reduce obesity, an important risk factor for DM. Therefore, we can conclude that it could be a non-invasive biomarker or a potential target for the treatment of patients with DR. Our results implied an opposite mutual causal relationship between *Eubacterium coprostanoligenes group* and DNP.

Although no studies of *Eubacterium coprostanoligenes group* were relevant to neuropathy, some studies indicated improvement of dyslipidemia induced by high-fat diet (74) and amelioration of fasting blood glucose, hemoglobin, serum levels of endotoxin, interlukin-6, tumor necrosis factor-α, and interlukin-1β in prediabetes (75). Hyperlipidemia and prediabetes are risk factors for DNP (66, 76). As a result, we recognize that *Eubacterium coprostanoligenes group* could possibly mediate the beneficial effects of DNP. MR analyses for diabetic microvascular complications on GM substantiated positive effects on the other GM. However, OR generally ranged from 0.9 to 1.1, demonstrating little causal relationship. These strains were still novel diagnostic biomarkers of diseases and therapeutic breakthrough.

Inevitably, it should be noted that our study has some limitations that could have affected the results. Firstly, the GWAS included only individuals of European descent; thus, the generation of our findings to other races is limited. Secondly, because the summary statistics instead of raw data were utilized in the analysis, microvascular complications caused by T1DM or T2DM were lack of specific information in the FinnGen database, which restricted the further subgroup analyses. Thirdly, a relatively lenient GWAS significance threshold (*p* < 1e−05) was set for more genetic variations in IVs in order to perform horizontal pleiotropy detection and sensitivity analysis. Thus, FDR correction was utilized to lower the probability of being false positive. Fourthly, GM together with its metabolites and by-products played important roles on the onset and progression of diabetic microvascular complications. However, our study only explored the causal relationship between microvascular complications and GM. Therefore, it would be helpful to perform causal association between GM and diabetic microvascular complications in diverse European and non-European populations for more generalizability. Multivariable MR analysis can also be considered for the corresponding metabolites in future endeavors. Lastly, although amplicon sequence variant-level analysis provided better resolution and more accurate results in the 16S rRNA gene-based microbiota studies in comparison with taxa-level analysis, we only obtained datasets of taxa.

## Conclusion

The present study gave credence to the concept that GM may be a promising therapy in diabetic microvascular complications. Further experiments of GM therapy on diabetic microvascular complications are warranted to elucidate their effects and specific mechanisms.

## Data availability statement

The original contributions presented in the study are included in the article/**Supplementary Material**. Further inquiries can be directed to the corresponding author.

## Ethics statement

All the GWAS summary statistics of diabetic complications in this study were acquired from the FinnGen research project (https://r9.finngen.fi/). The studies were conducted in accordance with the local legislation and institutional requirements. Written informed consent for participation was not required from the participants or the participants’ legal guardians/next of kin. The summary-level data involved in this study are free of identified and publicly available for download. Respective institutions have approved the ethics statement of Each GWAS in this study. There was no individual-level data in this study, thus, new ethical review board approval was unnecessary.

## Author contributions

PZ: Conceptualization, Methodology, Validation, Visualization, Writing – original draft, Writing – review & editing. ZH: Data curation, Software, Writing – original draft. WX: Methodology, Writing – original draft. YC: Formal analysis, Writing – review & editing. ZZ: Supervision, Visualization, Writing – review & editing. JY: Funding acquisition, Supervision, Validation, Writing – review & editing, Conceptualization.

## Supplementary material

Supplementary Figure 1 Scatter plots for the causal association between **(A)** *Bacteroidetes*, **(B)** *Bacteroidia*, **(C)** *Verrucomicrobiae*, **(D)** *Bacteroidaceae*, **(E)** *Victivallaceae*, **(F)** *Catenibacterium*, **(G)** *Coprococcus2*, **(H)** *Lachnoclostridium*, **(I)** *Lactococcus*, and **(J)** *Parasutterella* and DKD. The slope of each line corresponds to the estimated MR effect in different models.

Supplementary Figure 2 Scatter plots for the causal association between **(A)** *Bacteroidia*, **(B)** *BacteroidalesS24_7group*, **(C)** *ClostridialesvadinBB60group*, **(D)** *Peptostreptococcaceae*, **(E)** *Eubacterium nodatum group*, **(F)** *Actinomyces*, **(G)** *Eisenbergiella*, **(H)** *Olsenella*, **(I)** *Parasutterella*, **(J)** *RuminococcaceaeUCG003*, and **(K)** *RuminococcaceaeUCG011* and DR. The slope of each line corresponds to the estimated MR effect in different models.

Supplementary Figure 3 Scatter plots for the causal association between **(A)** *Acidaminococcaceae*, **(B)** Peptococcaceae, **(C)** *Eubacterium coprostanoligenes group*, **(D)** *Alistipes*, **(E)** *ChristensenellaceaeR_7group*, **(F)** *Eggerthella*, and **(G)** *RuminococcaceaeUCG013* and DNP. The slope of each line corresponds to the estimated MR effect in different models.

Supplementary Figure 4 MR leave-one-out sensitivity analysis for **(A)** *Bacteroidetes*, **(B)** *Bacteroidia*, **(C)** *Verrucomicrobiae*, **(D)** *Bacteroidaceae*, **(E)** *Victivallaceae*, **(F)** *Catenibacterium*, **(G)** Coprococcus2, **(H)** *Lachnoclostridium*, **(I)** *Lactococcus*, and **(J)** *Parasutterella* on DKD. Calculate the MR results of the remaining IVs after removing the IVs one by one.

Supplementary Figure 5 MR leave-one-out sensitivity analysis for **(A)** *Bacteroidia*, **(B)** *BacteroidalesS24_7group*, **(C)** *ClostridialesvadinBB60group*, **(D)** Peptostreptococcaceae, **(E)** *Eubacterium nodatum group*, **(F)** Actinomyces, **(G)** Eisenbergiella, **(H)** Olsenella, **(I)** *Parasutterella*, **(J)** *RuminococcaceaeUCG003*, and **(K)** *RuminococcaceaeUCG011* on DR. Calculate the MR results of the remaining IVs after removing the IVs one by one.

Supplementary Figure 6 MR leave-one-out sensitivity analysis for **(A)** *Acidaminococcaceae*, **(B)** Peptococcaceae, **(C)** *Eubacterium coprostanoligenes group*, **(D)** *Alistipes*, **(E)** *ChristensenellaceaeR_7group*, **(F)** *Eggerthella*, and **(G)** *RuminococcaceaeUCG013* on DNP. Calculate the MR results of the remaining IVs after removing the IVs one by one.
