# Supplementary figures and images for: Association between gut microbiota and diabetic microvascular complications: a two-sample Mendelian randomization study
Source: Front Endocrinol (Lausanne). 2024 Aug 2;15:1364280. doi: 10.3389/fendo.2024.1364280 (PMC11327146; doi:10.3389/fendo.2024.1364280)

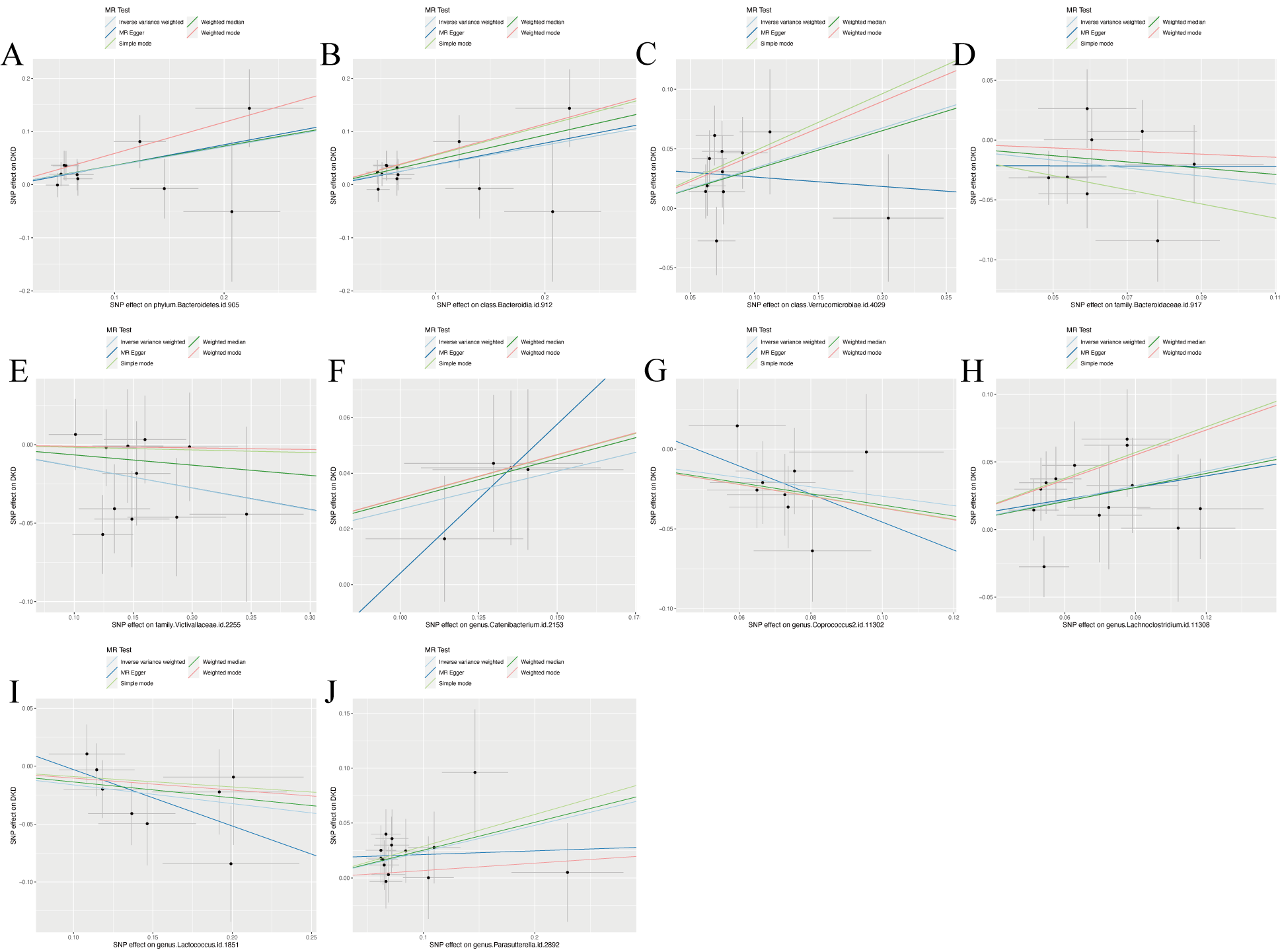

Supplement: Supplementary Figure 1 — Scatter plots for the causal association between (A) Bacteroidetes, (B) Bacteroidia, (C) Verrucomicrobiae, (D) Bacteroidaceae, (E) Victivallaceae, (F) Catenibacterium, (G) Coprococcus2, (H) Lachnoclostridium, (I) Lactococcus, and (J) Parasutterella and DKD. The slope of each line corresponds to the estimated MR effect in different models. [file Image_1.tif]

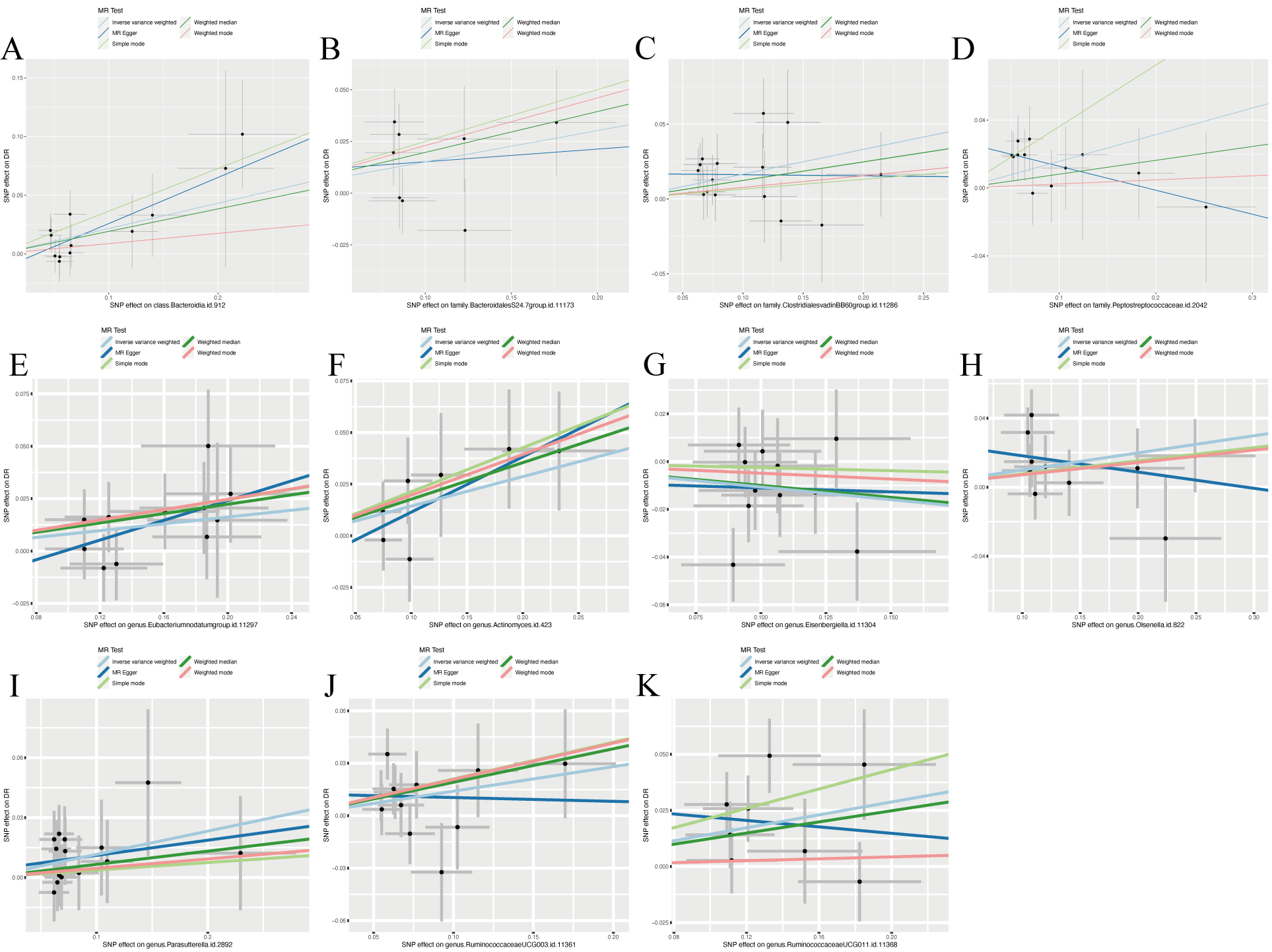

Supplement: Supplementary Figure 2 — Scatter plots for the causal association between (A) Bacteroidia, (B) BacteroidalesS24_7group, (C) ClostridialesvadinBB60group, (D) Peptostreptococcaceae, (E) Eubacterium nodatum group, (F) Actinomyces, (G) Eisenbergiella, (H) Olsenella, (I) Parasutterella, (J) RuminococcaceaeUCG003, and (K) RuminococcaceaeUCG011 and DR. The slope of each line corresponds to the estimated MR effect in different models. [file Image_2.tif]

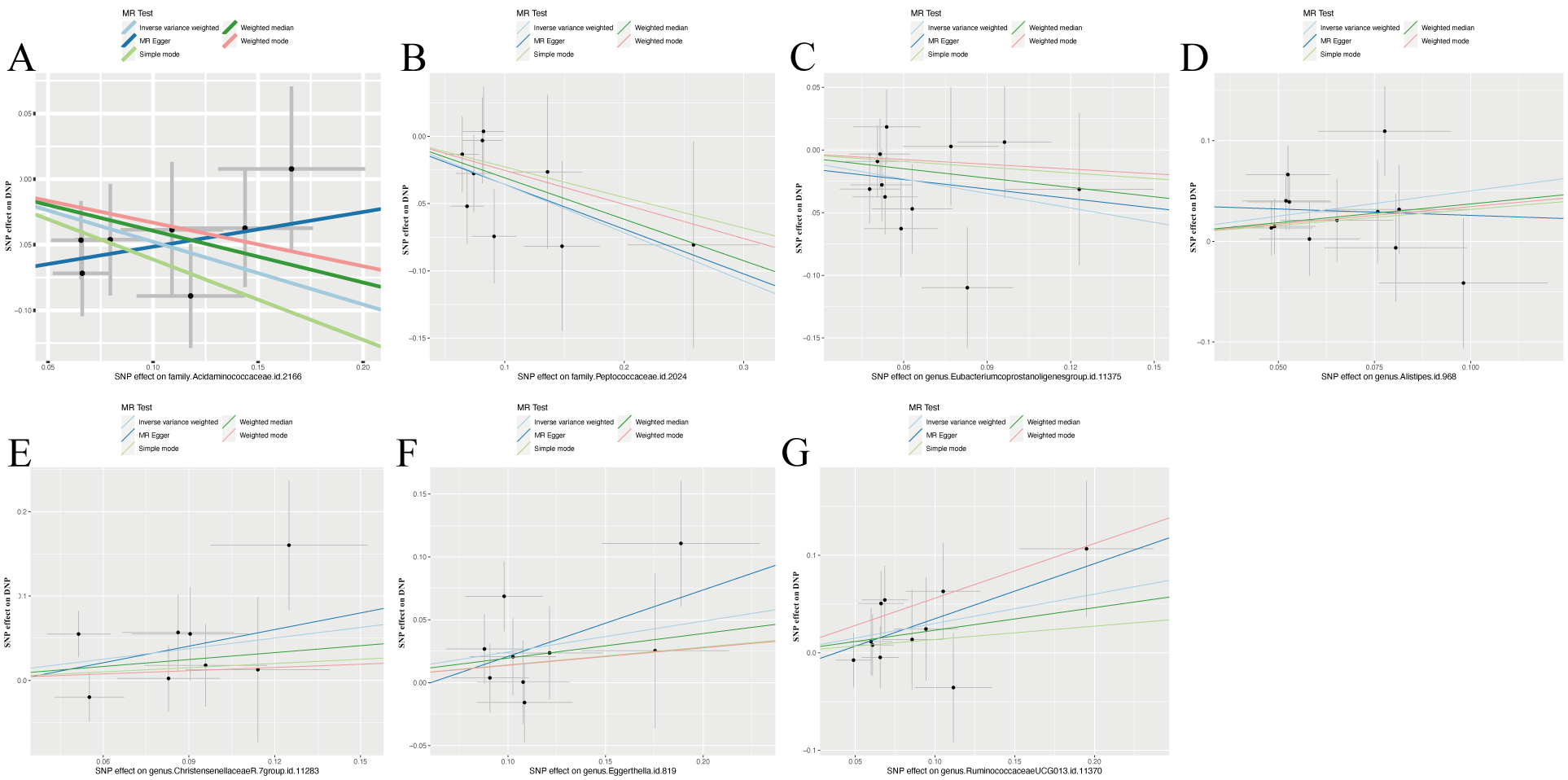

Supplement: Supplementary Figure 3 — Scatter plots for the causal association between (A) Acidaminococcaceae, (B) Peptococcaceae, (C) Eubacterium coprostanoligenes group, (D) Alistipes, (E) ChristensenellaceaeR_7group, (F) Eggerthella, and (G) RuminococcaceaeUCG013 and DNP. The slope of each line corresponds to the estimated MR effect in different models. [file Image_3.tif]

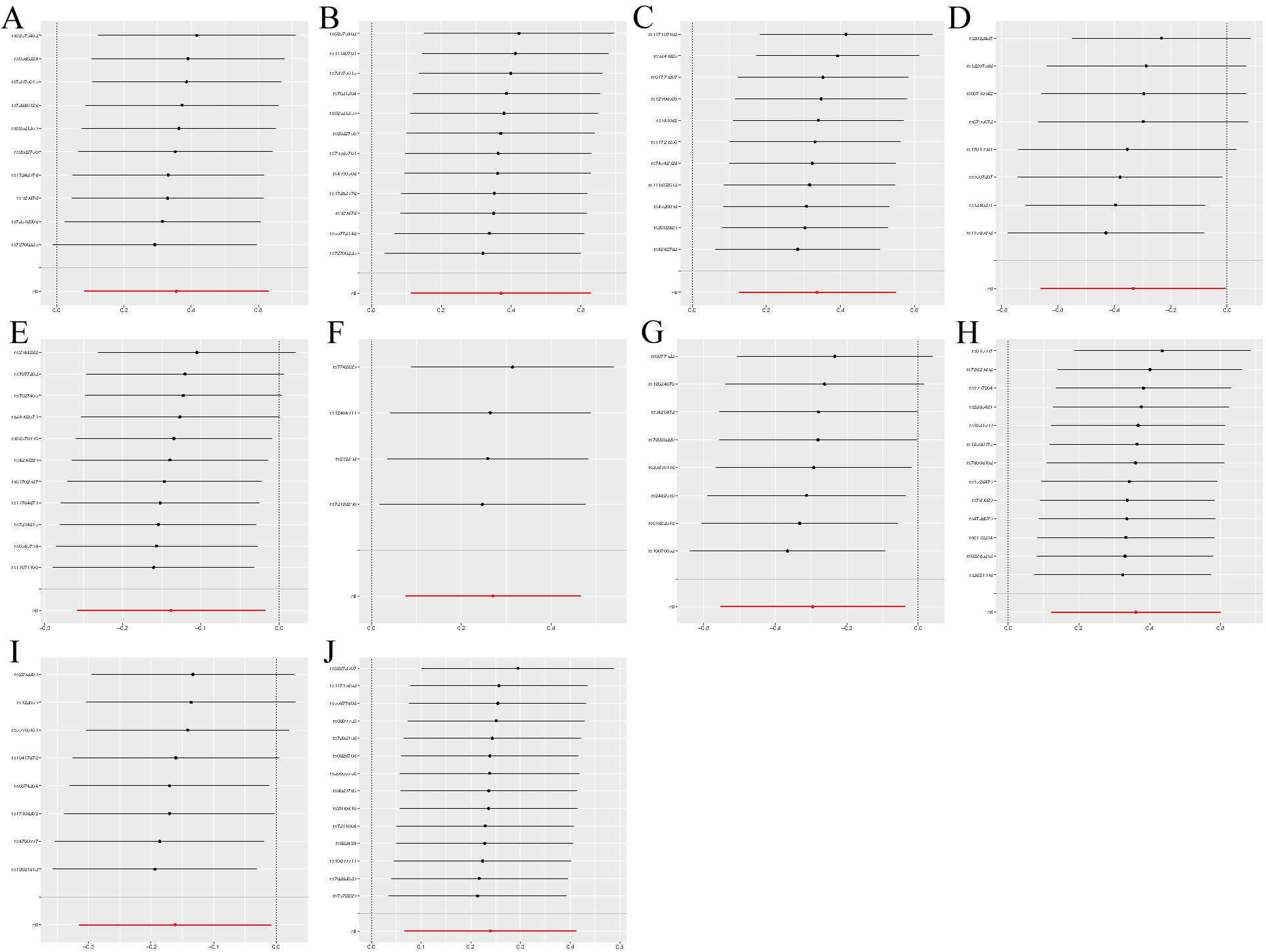

Supplement: Supplementary Figure 4 — MR leave-one-out sensitivity analysis for (A) Bacteroidetes, (B) Bacteroidia, (C) Verrucomicrobiae, (D) Bacteroidaceae, (E) Victivallaceae, (F) Catenibacterium, (G) Coprococcus2, (H) Lachnoclostridium, (I) Lactococcus, and (J) Parasutterella on DKD. Calculate the MR results of the remaining IVs after removing the IVs one by one. [file Image_4.tif]

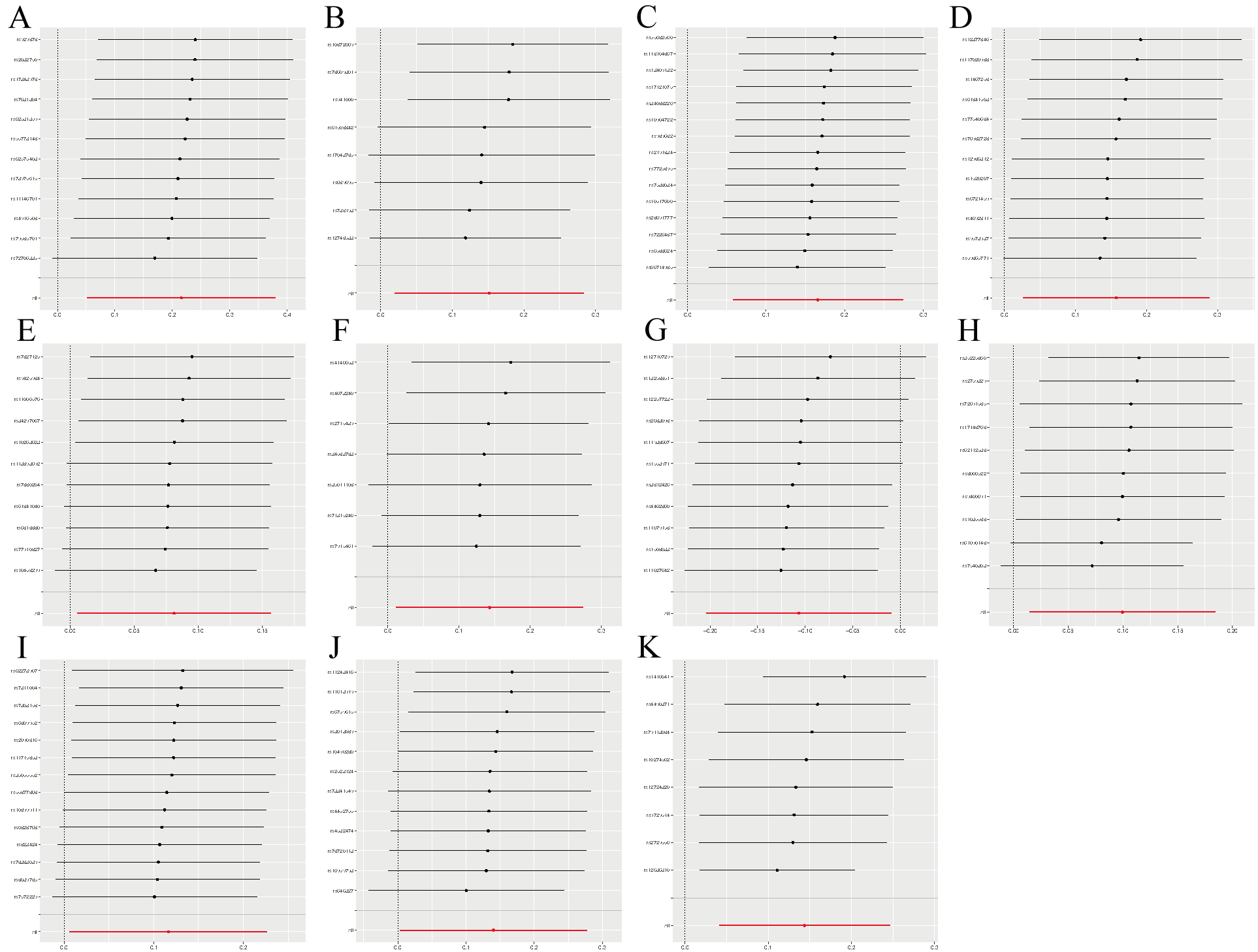

Supplement: Supplementary Figure 5 — MR leave-one-out sensitivity analysis for (A) Bacteroidia, (B) BacteroidalesS24_7group, (C) ClostridialesvadinBB60group, (D) Peptostreptococcaceae, (E) Eubacterium nodatum group, (F) Actinomyces, (G) Eisenbergiella, (H) Olsenella, (I) Parasutterella, (J) RuminococcaceaeUCG003, and (K) RuminococcaceaeUCG011 on DR. Calculate the MR results of the remaining IVs after removing the IVs one by one. [file Image_5.tif]

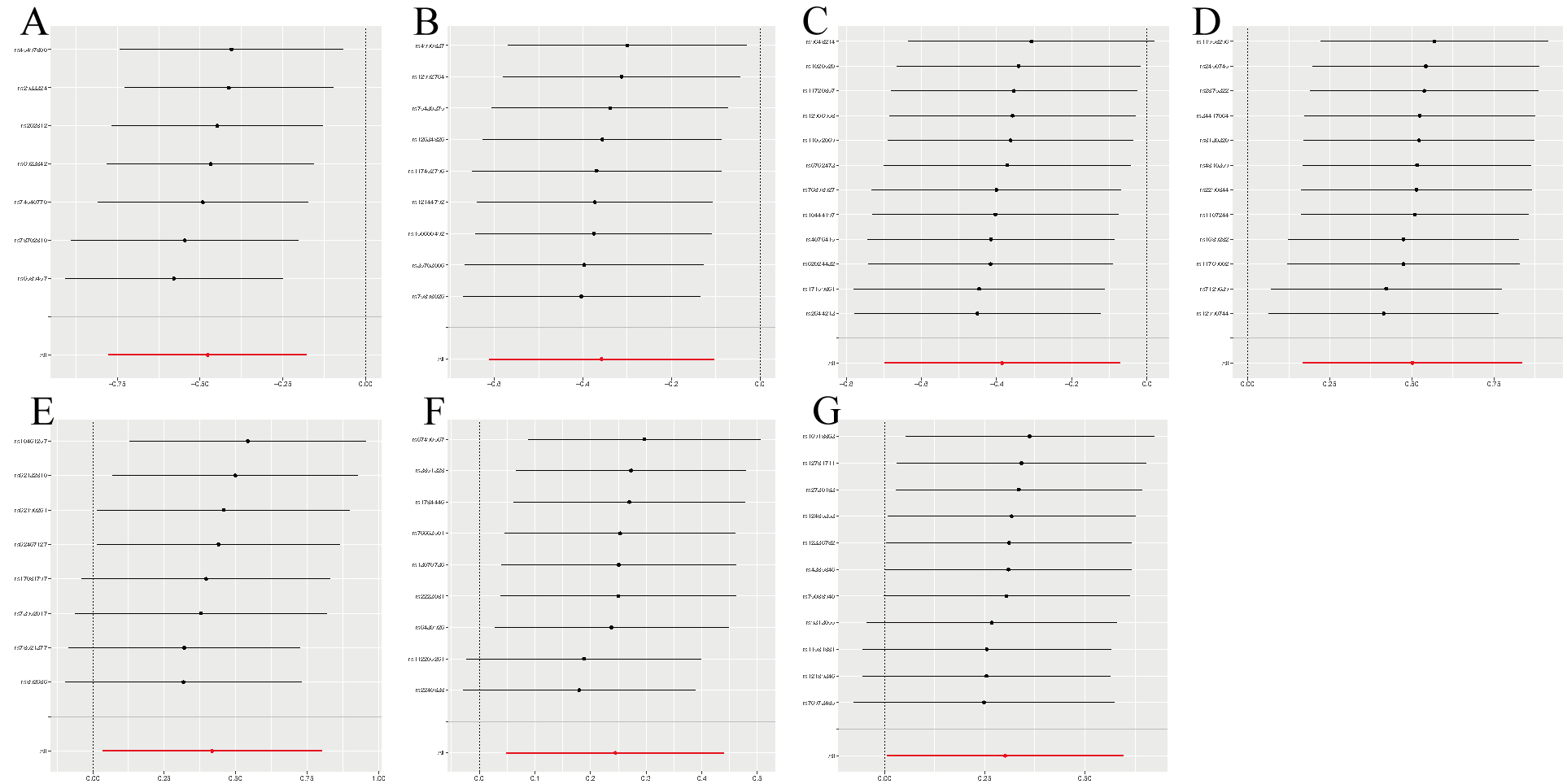

Supplement: Supplementary Figure 6 — MR leave-one-out sensitivity analysis for (A) Acidaminococcaceae, (B) Peptococcaceae, (C) Eubacterium coprostanoligenes group, (D) Alistipes, (E) ChristensenellaceaeR_7group, (F) Eggerthella, and (G) RuminococcaceaeUCG013 on DNP. Calculate the MR results of the remaining IVs after removing the IVs one by one. [file Image_6.tif]
